# Food insecurity arises the likelihood of hospitalization in patients with COVID-19

**DOI:** 10.1038/s41598-021-99610-4

**Published:** 2021-10-08

**Authors:** Mohammad Ariya, Jalal Karimi, Somayeh Abolghasemi, Zeinab Hematdar, Mohammad Mehdi Naghizadeh, Maryam Moradi, Reza Barati-Boldaji

**Affiliations:** 1 Noncommunicable Diseases Research Center, Fasa University of Medical Sciences, Fasa, Iran; 2 Department of Nutrition, Fasa University of Medical Sciences, Fasa, Iran; 3 Department of Infectious Diseases, School of Medicine, Fasa University of Medical Science, Fasa, Iran; 4 Nutrition Research Center, Shiraz University of Medical Sciences, Shiraz, Iran

**Keywords:** Infectious diseases, Health policy, Nutrition, Public health, Quality of life

## Abstract

The World Health Organization (WHO) has declared the Corona pandemic as a public health emergency. This pandemic affects the main pillars of food security. This study aimed to investigate the relationship between food insecurity and the probability of hospitalization and the length of the recovery period after getting COVID-19. The cross-sectional study was performed through the census on COVID-19 patients diagnosed in Fasa, Iran. Informed consent, demographic, and food security questionnaire were completed over the phone. Then, all patients were followed up until recovery. Data were analyzed using SPSS26 and Chi-square test, t-test, and logistic regression (*P* < 0.05). In this study, 219 COVID-19 patients [100 (54.7%) male and 119 (54.3%) female] with a mean age of 40.05 ± 15.54 years old were examined. Possibility of hospitalization and the length of the recovery period of more than one month was significantly longer in the food-insecure group (*P* = 0.001) and (*P* = 0.37), respectively, but the mean length of hospital stay in the two groups was not significantly different (*P* = 0.76). After adjusting for all confounding variables, people with food insecurity were 3.9 times more likely to be hospitalized than those with food security. Overall, we observed that food-insecure people were significantly more likely to be hospitalized than the secure group.

## Introduction

In December 2019, a series of pneumonia appeared in China, which later revealed that this type of acute respiratory disease was a novel coronavirus (SARS-CoV-2), later known as COVID-19 worldwide^1^. The virus eventually spread around the world, and on March 11, 2020, the World Health Organization (WHO) declared it a pandemic^2^. WHO has declared COVID-19 as a public health emergency and international concern since February 1, 2020^3^.

COVID-19 has an extensive spectrum of severity. Some of these people may recover by receiving outpatient medical care and medication at home. But on the other hand, some may be hospitalized and be under emergency care. Some of them may not respond to treatment and lose their lives. Hospitalization is an important criterion because it determines the strain of a pandemic on the health care system^4^. As a result, estimates of the burden and severity of COVID-19 disease and the rate of hospitalization in society, especially in food-insecure groups, are crucial to recognizing appropriate intervention strategies and healthcare plans^4^. In addition, with a clear understanding of the relationship between food insecurity and hospitalization, it may be possible to predict the number of people to be hospitalized. This matter may ensure the health system's sustainability throughout the duration of the coronavirus pandemic.

The COVID-19 pandemic, like other pandemics, can cause significant changes around the world that affect all countries, cities, and villages, it may ultimately lead to changes in lifestyle and food choices and affect food security and access to food for different groups in society^5^. Among these, families with children under 6, female-headed households, adults living alone, people with disabilities, and low-income families are most affected by food insecurity during COVID-19 and also experience food insecurity more than others^6^.

Food insecurity can be defined as "uncertain or limited availability of adequate and healthy food or uncertain or limited ability to acquire acceptable foods in socially acceptable ways"^7^. COVID-19 pandemic affects the four main pillars of food security (availability, access, utilization, stability)^8^. In other words, the pandemic directly and severely affects food access, food availability, or distribution, and these matters shift consumer demand towards cheaper and less nutritious foods, as a result, it endangers food stability and utilization^8^.

Apart from the categories mentioned above, issues such as the quality of the diet, the stability of access to food sources over time and the distance to the source of the food production are also important factors in food security, and the recent pandemic has a potential impact on all of these cases^9^. Besides, this pandemic has pushed up food prices and is expected to increase food prices in most countries as this trend continues^10^. Therefore, in such circumstances, global food insecurity as an important issue should be paid as much attention as possible^11^.

Food and Agriculture Organization of the United Nations (FAO) in 2017 published a report which indicates Asian countries are confronted with the highest rate of food insecurity, after African countries^12^. In this regard, other studies stated that the range of food insecurity in each family are so varied and this matter could be changed from anxiety of food availability to severe starvation, especially in children with no access to food^7^. It is noteworthy to mention, income is a vital issue in obtaining food security, and if a family spends more than 75 percent of their income spend to purchase food, they categorize as the highest level of food insecurity family^13^. It seems that the current pandemic worse this situation, especially in Asian and African countries.

Undoubtedly, having a clear understanding of whether in the arisen crisis, food insecurity can lead to hospitalization or not and whether the speed of recovery from the disease in people with food security will increase or not can help control the recent pandemic as much as possible. Furthermore, knowing the level of household food security and its short-term or long-term impact on the coronavirus pandemic may be a way to reduce the burden of disease and improve food security in the community through government assistance to vulnerable households. Therefore, due to the limited studies in this field, we decided to conduct the present study to correlate the relationship between food security and the possibility of hospitalization and the length of the recovery period after getting the COVID-19 disease through telephone interviews in the city of Fasa, Iran.

## Results

In this study, 230 patients with COVID-19 were studied, 4 patients were reluctant to cooperate, 5 were under 18 years old, and 2 died before the end of the study, so the data of 219 patients with COVID-19 were examined (supplementary Fig. 1). Of these, 100 (54.7%) were male and 119 (54.3%) were female, with a mean age of 40.05–15.54 years and a mean BMI of 24.91. 4.52. Among these people, 64 (29.2%) lived alone, 123 (56.2%) were without any fixed income, 70 (32.0%) had less than a diploma and 35 (16%) were smokers. Details of the general characteristics of these people are listed in Table 1.

**Table 1:** Food insecurity and demographic information in the study population (n = 219).

		Total		Food insecurity				
				Food security		Food insecurity		*P*-value
		Number	Percent (%)	Number	Percent (%)	Number	Percent (%)	
Gender	Male	100	45.7	88	88.0	12	12.0	0.37
	Female	119	54.3	109	91.6	10	8.4	
Age (years)	≤ 50	154	70.3	141	91.6	13	8.4	0.22
	> 50	65	29.7	56	86.2	9	13.8	
Marital status	Single	64	29.2	56	87.5	8	12.5	0.43
	Married	155	70.8	141	91.0	14	9.0	
Occupational status	Stable income source	57	26.0	56	98.2	1	1.8	0.04
	Unstable income source	39	17.8	33	84.6	6	15.4	
	Without income	123	56.2	108	87.8	15	12.2	
Education	Less than high school's diploma	70	32.0	54	77.1	16	22.9	
	High school's diploma	80	36.5	74	92.5	6	7.5	< 0.001
	Bachelor's degree and higher	69	31.5	69	100.0	0	0.0	
Address	Urban	156	71.2	145	92.9	11	7.1	0.02
	Rural	63	28.8	52	82.5	11	17.5	
Household income (per year)	≤ 1200 $/year	22	10.0	13	59.1	9	40.9	< 0.001
	> 1200 $/year	197	90.0	184	93.4	13	6.6	
Smoking	Yes	35	16.0	28	80.0	7	20.0	0.03
	No	184	84.0	169	91.8	15	8.2	
BMI (kg/m^2^)	< 25	125	57.1	114	91.2	11	8.8	0.47
	> 25	94	42.9	83	88.3	11	11.7	
Number of Household Members	Single	25	11.4	20	80.0	5	20.0	
	≤ 5	147	67.1	133	90.5	14	9.5	0.17
	> 5	47	21.5	44	93.6	3	6.4	

	Mean	SD	Mean	SD	Mean	SD	*P*-value	
Age (years)	40.05	15.54	39.30	15.33	46.73	16.19	0.03	
BMI (kg/m^2^)	24.91	4.52	24.80	4.49	25.87	4.72	0.29	

BMI; Body mass index.

The findings of this study showed that the average score of food security in the study population was 0.85 ± 2.59, thus 22 people (10%) of the population had food insecurity while the rest of the population enjoyed food security. Table 1 also shows the factors affecting food security. Food insecurity in people without income and with variable income (*P* = 0.04), low literacy (*P* < 0.001), rural (*P* = 0.02), with poor economic status (*P* < 0.001) and smokers (*P* = 0.03) were more than other people. The mean age of the food insecurity group was higher than the others (*P* = 0.03) but the mean body mass index in the two groups was not significantly different (*P* = 0.29).

Fever was the most common symptom among patients (111 patients (50.7%)). On the other hand, eye redness (19 patients (8.7%)) had the lowest frequency among these symptoms. More details are given in Table 2.

**Table 2:** Signs and symptoms of the study population.

	NO		YES	
	Number	Percent (%)	Number	Number (%)
Fever	108	49.3	111	50.7
Headache and body aches	112	51.1	107	48.9
Cough	140	63.9	79	36.1
Taste and smell disorders	150	68.5	69	31.5
Shortness of breath	156	71.2	63	28.8
Vomiting and diarrhea	171	78.1	48	21.9
Sore throat	174	79.5	45	20.5
Runny nose	187	85.4	32	14.6
Eye redness	200	91.3	19	8.7

Among people with COVID-19 disease who faced food insecurity 12 (54.5%), and in the group with food security 42 (21.3%) were hospitalized, which is a statistically significant difference (*P* = 0.001), but the mean length of hospital stay in the two groups was not significantly different (*P* = 0.76). Besides, the duration of recovery between the two groups with and without food insecurity showed a significant difference (*P* = 0.01) and in food-insecure people, the recovery time was significantly longer than one month. However, the mean length of recovery did not differ between the two groups (*P* = 37/0) (Table 3).

**Table 3:** Hospitalization and recovery in the study population.

		Status of food insecurity				
		Food safe		Food insecurity		
		Number	Percent (%)	Number	Percent (%)	*P*-value
Hospitalization	No	155	78.7	10	45.5	0.001
	Yes	42	21.3	12	54.5	
Duration of Recovery	< 30 days	185	93.9	17	77.3	0.01
	> 30 days	12	6.1	5	22.7	

	Mean	SD	Mean	SD		
Length of hospitalization	8.57	6.18	8.00	3.88	0.76	
Length of recovery	18.45	11.68	20.82	13.16	0.37	

**Figure 1:**
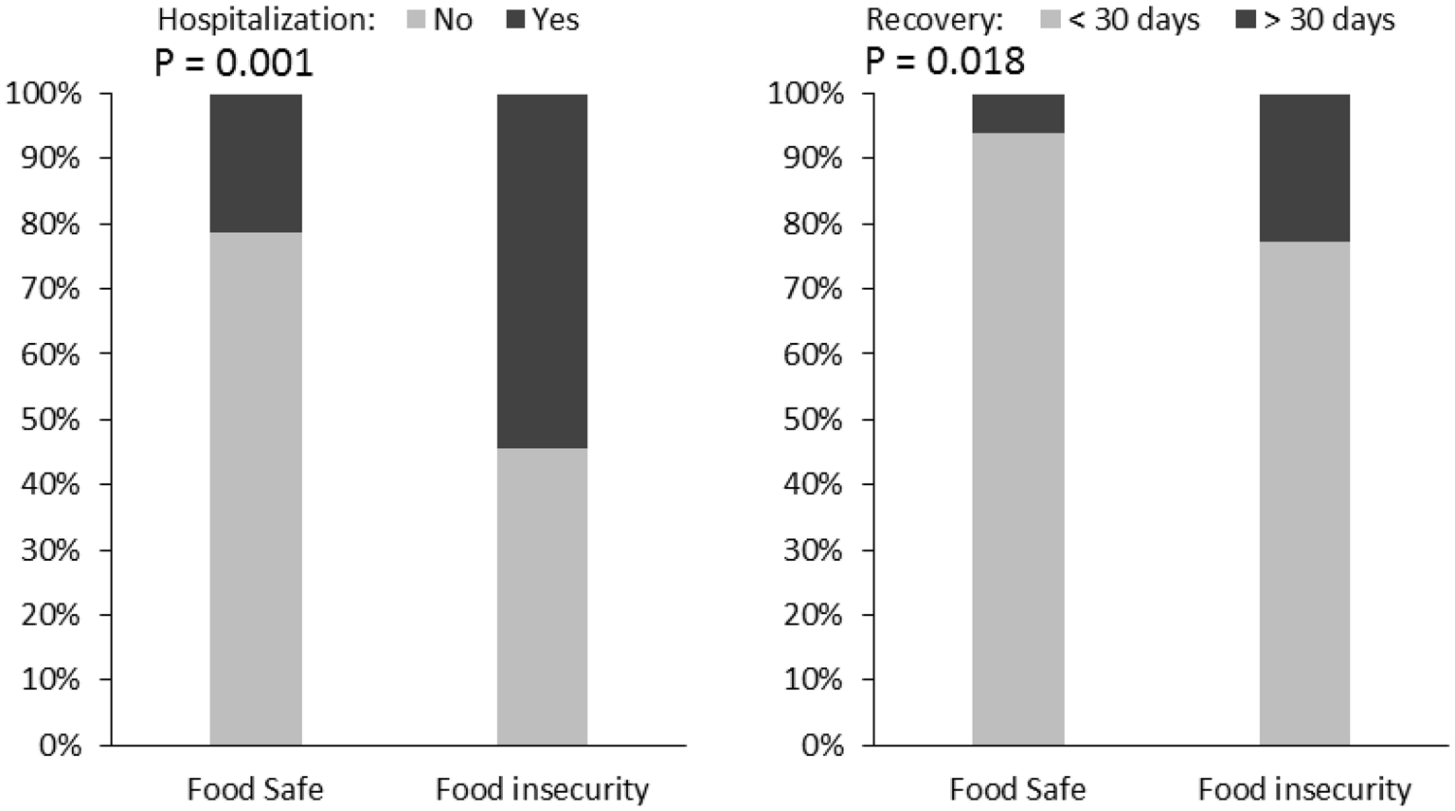
Hospitalization and recovery in the study population.

In addition to Table 4, this relationship is also shown in Fig. 1. This figure shows that the risk of hospitalization and recovery time of more than 30 days in food insecure people are significantly higher than in the food-secure group.

**Table 4:** Effect of food insecurity on hospitalization with the uni- and multivariate logistic regression.

		P-value	OR	95% C.I. for EXP(B)	
				Lower	Upper
Univariate	Food insecurity	0.001	4.42	1.79	10.95
Multivariate	Food insecurity	0.01	3.93	1.30	11.90
	Male gender	0.28	1.63	0.66	4.03
	Age > 50 years	0.007	3.07	1.35	6.96
	Married	0.22	1.72	0.71	4.16
	Income	0.51			
	Income (unstable/stable)	0.87	0.91	0.27	3
	Income (without/stable)	0.36	1.70	0.54	5.33
	Education	0.86			
	Education (diploma/lower) *	0.61	1.26	0.50	3.14
	Education (Upper/lower)**	0.65	1.31	0.40	4.29
	Address (urban)	0.92	1.04	0.48	2.23
	Lower household Income	0.24	1.94	0.64	5.89
	Smokers	0.62	1.29	0.46	3.63
	BMI (> 25 kg/m^2^)	0.28	1.46	0.72	2.95
	Household members	0.75			
	Household members (< 5/single)	0.52	1.42	0.47	4.30
	Household members (> 5/single)	0.85	1.12	0.30	4.17
	Constant	0.001	0.029		

BMI; Body mass index.

*Less Than High School's Diploma versus High School's Diploma.

**High School's Diploma versus Bachelor's Degree and Higher.

Table 4 shows that the rate of hospitalization of food-insecure people with COVID-19 is 4.42 (95% CI 10.95 − 1.79) is higher than patients with food security. The confounding effects of the underlying variables were removed; it was found that the above relationship still exists.

## Discussion

The present study showed that food-insecure people with COVID-19 had a longer recovery time. Also, our findings showed that food insecurity significantly increases the likelihood of hospitalization, and after adjusting for all confounding variables, people with food insecurity are 3.9 times more likely to be hospitalized than those in the food-secure group. The present study is one of the few studies to examine food security in patients with COVID-19. Other studies have addressed food security in the general population.

In the present study, food-insecure individuals were significantly more likely to be hospitalized than the food-secure group, and this finding remained significant after adjusting for variables. Other studies show that people in food-insecure households (especially children) are significantly more likely to be hospitalized for infectious diseases^14^. Besides, studies cite reasons such as poor diet quality, medication non-adherence or not having enough money to buy medication, lack of control over some chronic diseases such as diabetes, as factors for more possibility to be hospitalized in food-insecure people^15^.

Food choices based on the level of food security can make a significant contribution to the prevention or progression of respiratory diseases. Research shows that the quality of food intake in patients with malnutrition or food insecurity is reduced due to the greater tendency of these people to consume western diets to supply their calories^16^. In the western diet, a large portion of foods is related to sugar, refined grains, and saturated fats, and on the other hand, this group consumes a small amount of fiber and unsaturated oils, which are very good for health^16^. Therefore, it can be said that this issue may be one of the reasons for the hospitalization of more people with food insecurity than the food-secure group in the present study.

Our findings show that lack of food security significantly increases the recovery time after getting COVID-19. Food insecurity is associated with poor diet quality on the one hand^16^ and on the other hand, people with food insecurity are more likely to develop chronic diseases such as diabetes^17^ and obesity^18^. These factors may be one of the reasons why food-insecure adults recover longer than food-secure ones. Also, food-insecure people may avoid medication because they do not have enough money for food^19^, which itself in the COVID-19 pandemic may worsen or prolong the symptoms of the disease.

Changes in the level and extent of food insecurity during the COVID-19 pandemic can have a significant impact on the consequences of this pandemic so that increasing levels of food insecurity are associated with mortality, morbidity, and disease burden in many non-communicable diseases^20^ and lack of food security can exacerbate and prolong the effects of COVID-19^6^. In addition, the total amount of energy received can be directly related to the duration of recovery, so that the WHO considers the amount of energy consumed between 2500 and 3400 kcal per person per day as a measure of healthy living^21^ but it seems that most people with food insecurity receive far fewer calories.

According to our findings, food insecurity was associated with low income or poor economic status, illiteracy, and rural living. Food insecurity and low incomes make people more vulnerable to coronavirus. Because on the one hand, these people cannot buy all the food they need in one place and this causes more travel and on the other hand, these people are more exposed to severe hunger crises because they do not have enough financial resources to buy sufficient food^22^. Also, in Iran, people with lower incomes usually live in rural areas.

The COVID-19 pandemic has limited all stages of the food supply chain, including processing, production, procurement, and distribution^10^. In addition, in the recent pandemic, business closures, social distancing policies, fear of shopping, and fear of going out to shop because of the risk of exposure to the virus have led to increased food insecurity^6,23^. Besides, food availability, which is one of the categories of food security, is disrupted due to the loss of all or part of the income and the fear of depletion of food stocks^24^. Food insecurity and changes in eating habits and behaviors in the short term obviously can have a significant impact on the health of society, especially children^23,24^. The COVID-19 pandemic complicated the strategies used by low-income families further to combat food insecurity, and in some cases, families were unable to maintain their food security^23^. The negative impact may last for years, especially in food insecure and low-income households^6^.

Studies in other countries have shown that the COVID-19 pandemic reduced working hours and income in many households. For example, 43% of American households reported losing their jobs or their salaries due to the pandemic. This percentage was even higher than 50% in lower-income households^25^. Moreover, this seems to be even worse in countries that had a higher percentage of food insecurity before the COVID-19 outbreak^6^.

Research has shown that income plays an important role in food choices, so that in middle- and low-income countries, poor people spend more than a quarter of their income on basic foods such as wheat, rice, and corn, while this figure was only 14% in non-poor families^8^. Besides, research shows that poor families spend about 50 percent of their income on non-essential foods such as fruits, vegetables, and animal proteins, and reduction of the revenue causes poor families to even give up consuming these food groups^8^. This reduces the dietary diversity in low-income families, as a result, the intake of micronutrients and antioxidants decreases, and eventually endangers their health status^8,26^. Therefore, choosing cheap foods and having an imbalanced diet in families with food insecurity, because these foods are high in fat and sugar, is itself a risk factor in the development of respiratory diseases^27^.

Having a balanced diet is an integral part of controlling risk management strategies in pandemics, and the recent pandemic is no exception^28^. One of the most frequent recommendations to prevent COVID-19 disease is the high intake of fruits and vegetables, because this food group is high in antioxidants, they are very effective in boosting the immune system^29^. In addition, the consumption of animal proteins during the recovery period of the disease is highly recommended, because it promotes faster recovery^29^. It is evident that a decrease in income level makes food-insecure people unable to consume fruits, vegetables, and proteins, and their food basket tends to consume cheap, high-calorie foods^30,31^. This has a significant effect on lowering the level of the immune system and thus worsening the disease and the duration of it in case of having COVID-19.

There is ample evidence that a balanced diet has a significant effect on the immune system and disease susceptibility. Meanwhile, studies have shown that certain nutrients are very efficient in the effective activity of the immune system, this mechanism may be caused by the activation of some cells, changes in the production of signaling molecules, and the effect on gene expression^32^. Therefore, deficiency of some macronutrients such as protein and some micronutrients such as iron, zinc, vitamins A, E, B6, B12, which mainly play an important role in maintaining the function of the immune system, in addition to low energy intake can reduce immune system activity and increases the likelihood of susceptibility to infection^33^.

On the other hand, having an unbalanced diet, in the long run, activates the innate immune system and inhibits the adaptive response of the immune system to increased oxidative stress, as a result, it causes a delayed response in the adaptive response of the immune system, which is considered as one of the most important strategies of the immune system against pathogens^16^. Therefore, it is recommended to improve food habits and security by having a balanced diet and avoid western diets it may be one of the most important ways to boost the immune system and control infectious respiratory diseases^34^.

Evidence suggests that food insecurity can lead to poor health outcomes by activating inflammatory pathways^35,36^. Food insecurity can independently increase the level of inflammatory factors such as C-reactive protein (CRP)^36^, IL-6, and tumor necrosis factor (TNF) receptor 1^35^. On the other hand, food insecurity itself is considered a powerful stress factor, as studies show, stress also causes an increase in inflammatory factors in the individual^37^. An increase in these factors may lead to an increase in the level of inflammation in the body, resulting in a late recovery of food insecure people with COVID-19^6^.

As far as economics is concerned, although the economies of developed and developing countries have been affected by the current pandemic, developing nations are particularly vulnerable to such distortions^38^. This matter is because least developed countries have the lower budgetary capacity to buffer shocks in comparison to richer ones. Moreover, according to the FAO, most developing economies because of their heavy reliance on the importation of staple foods, recently have encountered to economic downturn due to the current pandemic^39^.

There are many ways to alleviate the food crisis and food insecurity during the COVID-19 crisis, especially in developing and poorly developed countries. In this regard, suggested solutions including (1) closely monitor food prices and funding in the storage of food, distribution, and marketing systems, (2) support from national agricultural and local food production, (3) use of social safety nets to protect those who are the most vulnerable, including women, children, the elderly, and the poor, (4) investment to build a more resilient food system to prevent or contain a food crisis, (5) use of the international market to ensure food supply, (6) cooperation of international organizations such as the World Trade Organization, FAO, World Bank with less-developed nations to reduce the risk of food crises, (7) utilization of fortification and supplementation to diminish the risk of malnutrition during the food crisis, (8) using nutrition education as a vital factor for improving food security, (9) tax reductions for staple foods, and, (10) early warning systems for managing food crises circumstances^40,41^.

The present study had some limitations. First, due to the nature of the study (cross-sectional), the cause-and-effect relationship cannot be extracted. Second, in the present study, factors related to mental states such as stress and anxiety were not examined, which is suggested that since food insecurity plays an important role in people’s mental health, these factors be examined in future studies. Third, in this study, the interview was conducted by telephone, which in this type of interview, there was a possibility of reporting an error. Fourth, food insecurity was assessed using the USDA retrospective questionnaire, since this questionnaire examines the level of food security in the past year, there is a possibility of non-recall error. It is noteworthy mentioning other variables such as religious or cultural beliefs, geographical areas, and food patterns and etiquette that can influence food insecurity, so, we suggest that these factors consider in future studies.

One of the strengths of the present study is that, according to our research, this study is one of the first studies to investigate the relationship between food security with the probability of hospitalization and the length of the recovery period in patients with COVID-19. Also, the use of an 18-item USDA validated questionnaire (not a short-form version) to assess food security adds credibility to our study. The sample size examined in the present study also adds to the strength of the present study because the total sample size was definitely positive for COVID-19. It is suggested that prospective studies be conducted in the future to better understand the impact of the coronavirus pandemic on food security.

## Conclusion

The present study observed that food-insecure adults were significantly more likely to be hospitalized than the secure group, and also the duration of recovery was significantly higher in them. Naturally, having information about the level of household food security helps policymakers (governments and global health-related organizations) and medical staff (physicians, nurses, and researchers) to cure COVID-19 patients as quickly as possible and reduce the duration of the disease and provide more practical solutions. Furthermore, making sure that food is available and accessible to those who need it ensures that essential nutrients are available to all segments of society to strengthen the immune system, and maybe this is a way to reduce the burden of the disease in society. It is obvious that the current state of long-term or short-term support for food insecure or at-risk individuals by governments and charities is helping a great deal to reduce the likelihood of hospitalization and speed up patient recovery.

## Methods

The present cross-sectional study examined the relationship between food insecurity and the duration of hospitalization and the duration of full recovery after getting the COVID-19 disease in patients covered by Fasa University of Medical Sciences who have been definitively diagnosed as positive in 2020. In the present study, all those who were definitively infected with COVID-19 based on diagnostic tests in the city of Fasa up to date 24 May 2020 participated in the study. The research was started on 20 February and ended on 27 May 2020 and because all subjects with COVID-19 could be followed (230 subjects), all patients were examined by the census.

In the present study, due to the definitive diagnosis of COVID-19 in participants and to comply with health principles, all interviews were conducted by telephone. First, after explaining the purpose of the project, the informed consent form was read through the telephone interview method for the study population, and if they wished to participate in the study, written informed consent was obtained from all of them. Afterward, the phone number and address of the project manager were provided to the subject so that he could easily contact the project manager if he had any questions. It should be noted that people who were unable to respond due to old age, illiteracy, or acute illness, their information completed through one of their first-degree relatives who lived with the sick ones or had accurate information about their life situation.

After receiving the informed consent form, first, the demographic questionnaire and then the food security questionnaire were asked from the patient by a trained person in a special order. The demographic questionnaire included factors such as age, sex, place of residence, level of education, height, and weight. After completing the questionnaire through the self-declaration of the interviewee, body mass index (BMI) was obtained by dividing weight (Kg) by height squared (m^2^). In this study, food insecurity was assessed by an 18-item the United States Department of Agriculture (USDA) Questionnaire, which has already been validated in Iran ^17^. Also, in the present study, all patients were followed up after the first contact to determine the duration of hospitalization and the duration of complete recovery (after the negative diagnostic tests). It should be noted that information such as symptoms was extracted from patients' medical records.

Inclusion criteria include: people over 18 years old, all people with the COVID-19 disease referred to one of the centers or hospitals under the auspices of the Fasa University of Medical Sciences with a definite positive test result, and exclusion criteria include: unwillingness to participate in the study, excessive disability to respond, mental illness psychosis, multiple sclerosis, and diseases leading to lack of recall (such as Alzheimer's) and unclear recovery status at the end of the study, were considered.

It is noteworthy to mention that the study protocol was following the Declaration of Helsinki guidelines and was approved by the Institutional Review Board (IRB) of the Ethical Committee of Fasa University of Medical Sciences (Ethical code: IR.FUMS.REC.1399.067).

### Measuring food security status

Food security status was assessed using the USDA 18-item Household Food Security Questionnaire as a valid questionnaire for epidemiological studies. It should be noted that in this study, the validated Persian version of this questionnaire was used^17^.

The USDA Household Food Security Status 18-item questionnaire, based on the method of Gary Bickel et al.^28^, is based on the answers "often correct", "sometimes correct", "almost every month", "some Months" and "Yes" give a positive score (1 point) and the answers "Not correct", "Does not know or refuse", "Only 1 or 2 months", and "No" are given a zero score. Based on the number of positive answers obtained from the questionnaire, individuals are divided into 4 groups: food secure (score of the questionnaire between 0 and 2), food-insecure without hunger (score 3 to 7), food insecure with moderate hunger (score 8 to 12) And food insecurity with severe hunger (scores 13 to 18); In the present study, for better comparison, individuals were divided into two groups: food secure and food insecure (food insecure without hunger, moderate hunger, and severe hunger) and were analyzed.

### Statistical analysis

The findings were displayed as mean and standard deviation. Qualitative variables were compared between the two groups with and without food security using the Chi-square test. Quantitative variables were compared between the two groups using an independent t-test. Multivariate logistic regression was used to eliminate the effect of confounding variables. In this model, patient variables were entered as response variables, and disease-related variables along with contextual variables, and food security variables were entered as independent variables (details of multicollinearity and statistical power of analysis were presented in Supplementary information file 2). Significance and odds ratios (OR) were reported from this model along with a 95% confidence interval. All calculations were performed in IBM SPSS (IBM Corp. Released 2019. IBM SPSS Statistics for Windows, Version 26.0. Armonk, NY: IBM Corp). A probability value of less than 0.05 was considered significant.

### Ethical approval and consent to participate

The study protocol was following the Helsinki Declaration and was confirmed by the Ethics Committee of Fasa University of Medical Sciences (Approval Code: IR.FUMS.REC.1399.067). The participants were informed about the research objectives and a consent form through the phone interview was obtained from the subjects before starting the survey.

## Supplementary Information

Supplementary Information file 1: Figure 1- Participant flowchart.

Supplementary Information file 2: Multicollinearity and power of analysis.

Supplementary Information File 3: Original dataset.

## Data Availability

The dataset used is included as Supplementary Information file 3.

## Abbreviations

WHO: World Health Organization
FAO: Food and Agriculture Organization of the United Nations
BMI: Body Mass Index
USDA: United States Department of Agriculture
IRB: Institutional Review Board
OR: Odds Ratios
CRP: C-reactive Protein
TNF: Tumor Necrosis Factor
