# Author Correction: Food insecurity arises the likelihood of hospitalization in patients with COVID-19

**DOI:** 10.1038/s41598-022-15459-1

**Published:** 2022-06-27

**Authors:** Mohammad Ariya, Jalal Karimi, Somayeh Abolghasemi, Zeinab Hematdar, Mohammad Mehdi Naghizadeh, Maryam Moradi, Reza Barati-Boldaji

**Affiliations:** 1 Noncommunicable Diseases Research Center, Fasa University of Medical Sciences, Fasa, Iran; 2 Department of Nutrition, Fasa University of Medical Sciences, Fasa, Iran; 3 Department of Infectious Diseases, School of Medicine, Fasa University of Medical Science, Fasa, Iran; 4 Nutrition Research Center, Shiraz University of Medical Sciences, Shiraz, Iran

Correction to: *Scientific Reports* 10.1038/s41598-021-99610-4, published online 08 October 2021

The original version of this article contained an error, as tests for multi-collinearity and power were not included.

Analyses rejecting the multi-collinearity problem and establishing the power of the study to be sufficient have now been included as Supplementary Information file 2.

In addition, the dataset for the study has now been included as Supplementary Information file 3.

Therefore, the Data Availability section,

“The datasets used and/or analyzed during the current study are available from the corresponding author on reasonable request to the corresponding author.”

now reads:

“The dataset used is included as Supplementary Information file 3”

And Supplementary information section,

“Supplementary Figure 1”

now reads:

“Supplementary Information file 1: Figure 1- Participant flowchart.

Supplementary Information file 2: Multicollinearity and power of analysis.

Supplementary Information file 3: Original dataset.”

Finally, under the subheading “Statistical Analysis”, in the Methods section, the text,

“In this model, patient variables were entered as response variables, and disease-related variables along with contextual variables, and food security variables were entered as independent variables.”

now reads:

“In this model, patient variables were entered as response variables, and disease-related variables along with contextual variables, and food security variables were entered as independent variables (details of multicollinearity and statistical power of analysis were presented in Supplementary Information file 2).”

The original Article has been corrected.

