# Supplementary figures and images for: Food insecurity arises the likelihood of hospitalization in patients with COVID-19
Source: Sci Rep. 2021 Oct 8;11:20072. doi: 10.1038/s41598-021-99610-4 (PMC8501085; doi:10.1038/s41598-021-99610-4)

**Supplementary Figure 1: Participant flowchart**

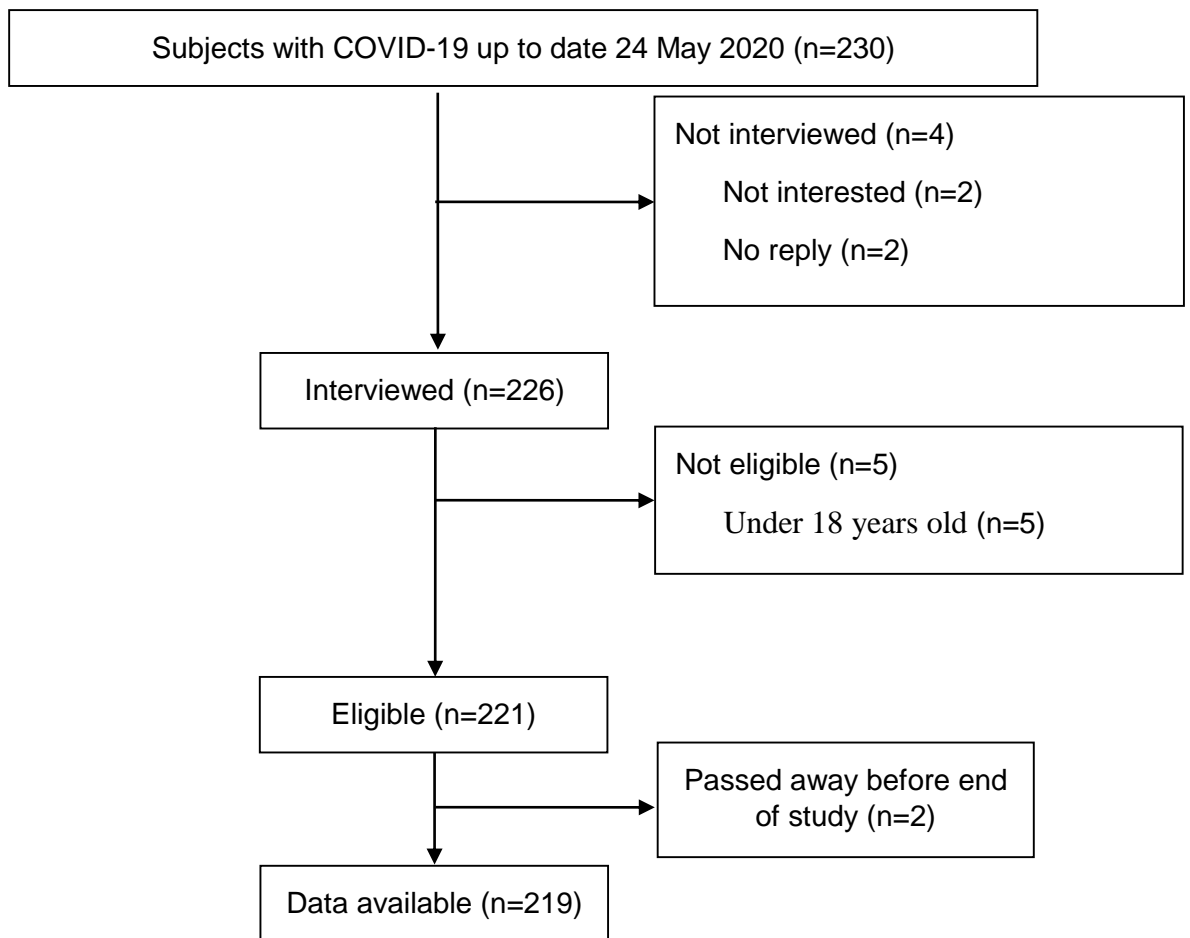

Supplement: Supplementary file 1 — Supplementary Information file 1: Figure 1- Participant flowchart. [file 41598_2021_99610_MOESM1_ESM.pdf]
