# Supplementary material for: Food insecurity arises the likelihood of hospitalization in patients with COVID-19
Source: Sci Rep. 2021 Oct 8;11:20072. doi: 10.1038/s41598-021-99610-4 (PMC8501085; doi:10.1038/s41598-021-99610-4)
Supplement: Supplementary file 2 — Supplementary Information file 2: Multicollinearity and power of analysis. [file 41598_2021_99610_MOESM2_ESM.docx]

Multicollinearity and power of the analysis

We analyzed our data for multicollinearity. The maximum Variance Inflation Score (VIF) of the variables in our regression model was 1.8 with an average of 1.3. We also, constructed another model with the food insecurity variable and 10 principal components of other predictors. In the new model, the odds ratio was 3.86 (95%CI: 1.28-11.64) and still be significant. Additionally, we did a few regression models and remove the predictors one by one to ensure the significance of the food insecurity variable. Theses analysis rejects the multicollinearity problem.

Our calculation showed that the power of our analysis is sufficient despite the unbalanced prevalence of food insecurity. The PASS software (PASS 15 Power Analysis and Sample Size Software (2017). NCSS, LLC. Kaysville, Utah, USA, ncss.com/software/pass.) calculated the power of analysis 0.89 in module. "Tests for the Odds Ratio in Logistic Regression with One Binary X (Wald Test)" with parameters: total sample size (N = 219), P-value in the univariate logistic regression (Alpha = 0.001), baseline probability (P0 or P (hospitalization = 1 | food insecurity = 0) = 42/155 = 0.21), Odds ratio (OR = 4.42), Prevalence of X (Px = 22/219 = 0.10). The calculated power of analysis comes from the univariate model and the multivariate model has more power, so, we don't worry about the power.
